# Predicting colorectal cancer risk in FAP patients using patient-specific organoids

**DOI:** 10.1038/s41417-025-00923-7

**Published:** 2025-07-22

**Authors:** Aline Habib, Rose Mamistvalov, Mira Malcov, Dalit Ben-Yosef

**Affiliations:** 1 The Fertility and IVF Institute, Lis Maternity Hospital, Tel-Aviv Sourasky Medical Center, Tel Aviv, Israel; 2 CORAL - Center of Regeneration and Longevity, Tel-Aviv Sourasky Medical Center, Tel Aviv, Israel; 3 Department of Cell and Developmental Biology, Sackler Faculty of Medicine, Sagol School of Neuroscience, Tel-Aviv University, Tel-Aviv, Israel

**Keywords:** Stem cells, Colorectal cancer

## Abstract

Colorectal cancer (CRC), a prevalent global cancer, is mostly sporadic. Familial adenomatous polyposis (FAP), arises from APC germline mutations. We established FAP-human embryonic stem cell lines (FAP1,2,3) with distinct APC mutations and differentiated them into colon organoids to study cancer development. While normal expressing APC lines and FAP3 formed complex organoids, FAP1,2 failed to differentiate. By utilizing CRISPR editing to correct APC mutations in FAP1,2, we succeeded in restoring their ability to form complex organoids expressing colon gene (CDX2). To elucidate the truncated APC proteins’ mechanism of action, we used AlphaFold2 algorithm to model their secondary structures. Structural analysis of the normal phenotype organoids (normal and FAP3) revealed 5-6 salt bridges only at the N-terminal oligomerization domain. In contrast, analysis of disease organoids-phenotype (FAP1,2) revealed a production of novel salt bridges, likely act in a dominant-negative manner on full-length APC, disrupting APC function and promoting tumorigenesis. Our study underscores the critical role of germline APC mutations in colon cancer initiation, revealing how specific mutations influence disease severity. By deciphering APC structure-function relationships, we illuminate potential therapies and the molecular underpinnings of APC mutations that precede clinical presentation.

## Scientific background

Colorectal cancer (CRC) is one of the most common cancers worldwide [1, 2]. Mutations that lead to loss of function of the tumor-suppressor gene adenomatous polyposis coli (APC) are found in 80-85% of sporadic CRC [3–7]. More than 5% of CRC cases are due to inherited disorders [7]. Familial adenomatous polyposis (FAP) and hereditary nonpolyposis colorectal cancer (HNPCC), both inherited in an autosomal dominant manner, are two major predisposition syndromes. Together, they account for approximately 1% and 5-7% of all CRC cases, respectively [8, 9]. FAP patients carry a germline mutation in one allele of APC and have a very high risk of acquiring an additional somatic mutation in the second allele of the gene, mainly in the colorectum. The acquisition of the second mutation will eventually lead to the development of hundreds to thousands of adenomas starting at a relatively young age, with 100% progression to CRC by the age of 40, if not treated [10, 11]. APC is considered the “gatekeeper” of colorectal carcinogenesis as mutations in this gene that lead to its loss of function trigger the adenoma-carcinoma-sequence, followed by accumulation of somatic mutations mainly in KRAS, SMAD4 and p53 [12–14]. The APC gene is located on chromosome 5 and consists of 8535 nucleotides (21 exons) [3]. It encodes a large multi-domain protein that negatively regulates Wnt signaling by controlling cellular levels of β-catenin.

Cells derived from FAP patients can serve as a good model to explore the genes and pathways involved in initiation of tumorigenic transformation in CRC in general and in FAP patients in particular [15]. We have previously derived three human embryonic stem cell (hESC) lines from FAP-affected embryos following preimplantation genetic diagnosis (PGD) [16, 17]. These hESC lines were derived from three different FAP families, and thus carry different heterozygous germline mutations in the APC gene. Importantly, these lines have not yet acquired the second hit, i.e. the somatic mutation in the second allele of the gene. For modeling CRC, we have already succeeded in differentiating these FAP-hESC lines into colon organoids demonstrating that the severity of the disease is related to the type and the location of the APC germline mutation, which varies between patients [16, 17], and affects the position of the somatic mutation. Most of the somatic mutations are in the mutation cluster region (MCR), resulting in C-terminus truncation of the protein [7, 11]. Thus, depending on the genetic background and epigenetics of CRC patients with similar histopathology, the disease may develop and progress in a completely different way [18]. Therefore, understanding the molecular pathways underlying the initiation and development of CRC is essential to identify novel molecular biomarkers for diagnosis, prognosis, and preventive medicine.

This study aimed to utilize CRISPR/Cas9-mediated targeted genome editing to rectify various APC germline mutations within FAP-hESCs. By doing so, we sought to elucidate the role these germline mutations play in determining disease severity.

## Methods

### Ethics

Genetically diagnosed diseased embryos for the derivation of hESC lines, were donated by patients following signing an informed consent, and the study of genetic disease was approved by the Israeli National Ethics Committee (7/04-043) and was conducted in accordance with the guidelines of the Bioethics Advisory Committee of the Israel Academy of Sciences and Humanities.

### hESC lines

Three FAP-hESC lines carrying germline mutations at different locations of the APC gene were previously derived in our lab following IVF-PGD treatment for FAP patients, were examined in this study: Lis25_FAP1 (FAP1); Lis30_FAP2 (FAP2) and Lis34_FAP3 (FAP3) [16, 19].

hESCs were cultured on wells coated with Geltrex (A1413202, ThermoFisher, Waltham, MA, USA), in mTeSR1 medium (85850, Stem Cell Technologies, Vancouver, Canada) supplemented with 100ug/ml Primocin (InvivoGen, San Diego, CA, USA) at 37⁰C with 5% CO_2_. hESCs were passaged at ~90% confluence using Accutase (SCR005, Stem Cell Technologies, Vancouver, Canada), and frozen in NutriFreez D10 (05-713-1B, Biological Industries, Kibbutz Beit-Haemek, Israel). After passaging or thawing, 10 µM ROCK inhibitor Y-27632 (#10005583, Cayman, Ann Arbor, MI, USA) was supplemented to mTeSR for the first 24 h to inhibit apoptosis.

### Generation of human colon organoids

Differentiation into colon organoids was induced as we described previously [17], with slight modifications. In summary, to generate definitive endoderm, hESCs were treated with 3 μM CHIR99021 (72054, Stem Cell Technologies, Vancouver, Canada) and 100 ng/ml activin A (120-14E, Peprotech, Cranbury, NJ, USA) in RPMI (Cellgro, Lincoln, NE, USA) medium supplemented with 2 mM GlutaMAX (Gibco, Thermo Fisher Scientific, Waltham, MA, USA) and 1X Pen/Strep (BioLab, Jerusalem, Israel), for one day. At the second day, cells were treated with 100 ng/ml activin A in RPMI supplemented with 2% FBS (S-FBSP-EU, SERANA, Pessin, Brandenburg, Germany), 2 mM GlutaMAX (35050-038, Gibco, Thermo Fisher Scientific, Waltham, MA, USA) and 100 U/ml Pen/Strep (03-033-1B Biological Industries, Kibbutz Beit-Haemek, Israel) for three days and 20% FBS on the third day. Cells were than subjected to hindgut differentiation by treatment with 3 μM CHIR99021 and 500 ng/ml FGF4 (100-31-500, Peprotech, Cranbury, NJ, USA) in RPMI supplemented with 1X B27 (17504044, Thermo Scientific, Waltham, MA, USA), GlutaMAX and 100 U/ml Pen/Strep for four days. From day 8, cells were cultured in a colonic medium comprised of advanced DMEM F12 (21331020, Thermo Scientific, Waltham, MA, USA) supplemented with 1X B27, 2 mM GlutaMAX, 100 U/ml Pen/Strep, 3 µM CHIR, 300 nM LDN (SM-1066208, Peprotech, Cranbury, NJ, USA) and 100 ng/ml EGF (AF-100-15-100, Peprotech, Cranbury, NJ, USA), and the medium was refreshed every two days. On day 20, cultures were disaggregated to single cells using Accutase for 10 min at 37⁰C and then re-suspended in 70 μl Matrigel (356231, Corning, Corning, NY, USA) drop-like structure, in Nunclon Delta Surface plates (177349, Thermo Scientific, Waltham, MA, USA). 10 µM of ROCK inhibitor was added for the first two days. Organoids were passed every 10 days (1:3).

### CRISPR/Cas9 plasmids

Two gRNAs targeting FAP1 and FAP2 mutation locus in the APC gene were designed using the Horizondiscovery.com algorithm for highly accurate and efficient functional gene knockout. FAP1 corr gRNA forward: CACCGAATGCTTGGTACTCATGATA and reverse: CTTACGAACCATGAGTACTATCAAA;

FAP2 corr gRNA forward: AAACGAGCCAGACAAACACTTTAGC and reverse CTCGGTCTGTTTGTGAAATCGCCAC.

gRNAs were prepared according to Zhang et al. [20] using clustered regularly interspaced short palindromic repeats (CRISPR)/CRISPR associated protein 9 (Cas9)-puromycin selection plasmid (pSpCas9n [BB]-2A-Puro [PX459] #62988, Addgene).

### Donor plasmid and mutagenesis

For generating the donor FAP1/2-pJET1.2 vectors, 1600 bp DNA fragments flanking each mutation area of FAP1 and FAP2 were amplified from human genomic DNA extracted from control hESCs. Subsequently, they were cloned into the pJET1.2 vector (#k1232, Thermo Scientific, Waltham, MA, USA) according to the manufacturer’s instructions. To avoid targeting by the designed Cas9-gRNA after the mutation correction, silent site direct mutations were introduced on the PAM sequence of the donor plasmid using the following primers:

FAP1corrSDM_forword: CAATGCTTGGgACgCAcGAcAAaGATGATATGTCGCGAACTTTG; FAP1corrSDM_reverse: GACATATCATCtTTgTCgTGcGTcCCAAGCATTGACAACAATGAATAC; FAP2corrSDM_forword: CTCTTACTTAtCGaAGCCAaACtAACACTTTAGCCATTATTGAAAGTGGAGG; FAP2corrSDM_reverse: GGCTAAAGTGTTaGTtTGGCTaCGaTAAGTAAGAGTGCCAACC.

### Plasmid transfection

CRISPR/Cas9 and the donor plasmid for directed mutation were transfected into FAP1-hESCs using Amaxa Nucleofector Technology, following a specific protocol for hESCs using P3 primary cell 4D Nucleofector X Kit (LNC-V4XP-3032, LONZA, Basel Switzerland). Rock inhibitor was added to the medium for 2 hours prior to dissociation. Cells were enzymatically harvested by Accutase and mechanical dissociation into single cells. In FAP2-hESCs transfection was carried out using Invitrogen Lipofectamine stem (STEM00003, Invitrogen, Thermo Scientific, Waltham, MA, USA). Cells were cultured until 30-40%. Confluence. 24 hours post-transfection, FAP1 and FAP2 cells were selected with 1 µg/ml Puromycin for 24 hours. Single-cell colonies from both FAP1 and FAP2 were propagated and analyzed for correction.

### Analysis of CRISPR/Cas9-Induced Mutation Correction

For determining mutation correction following CRISPR, DNA was extracted from FAP-hESCs using a Quick genomic DNA MiniPrep Kit (ZRD3025, ZYMO, Orange, CA, USA) according to the manufacturer’s instructions. For RFLP analysis, PCR amplification of each CRISPR/Cas9 target site was performed, the FAP1 primer was designed with a mutation for diagnose the WT allele of FAP1 locus by the restriction enzyme. FAP1-check forward: GACACGTGGAAATGGTGTATTCATTG and reverse: TGTAAAAGCTGGATGAGGAGAGGAAG; FAP2-check forward: TTGATAGCTACAAATGAGGACCCCAG and reverse: GAGTTAAACTAAGAGTAAGTAGTTATCTTTTCACAG. FAP1 PCR products were cleaved with NurI restriction enzyme (R3192S, New England Biolabs, Ipswich, MA, USA) and FAP2 products were cleaved with BsaJI (R0536S, New England Biolabs, Ipswich, MA, USA) restriction enzyme which diagnosed the WT allele. Candidates’ clones were also validated by Sanger sequencing.

### FACS analysis for the expression of pluripotent genes

hESC were dissociated into single cells with Accutase. For fluorescence-activated cell sorter (FACS) analysis, samples were pelleted and resuspended in FACS buffer (0.2% BSA ([#0332, VWR, Randor, PA, USA) in phosphate-buffered saline [PBS, 02-023, Biological Industries, Kibbutz Beit-Haemek, Israel]), followed by incubation with antibodies for pluripotent genes - Tra-1-60 (#330614, BioLegend, San Diego, CA, 1:25); SSEA4 (#330408, BioLegend, San Diego, CA 1:25) and EpCAM (#324218, BioLegend, San Diego, CA #324218, 1:25). Samples were analyzed by FACSCanto flow cytometer (BD Biosciences, Franklin Lakes, NJ, USA).

### Immunohistochemical analysis

hESCs were fixed with 4% paraformaldehyde (PFA, P6148, Sigma-Aldrich, Burlington, MA, USA). For intracellular staining, cells were incubated in blocking solution (2.5% BSA in PBS) with 0.1% Triton, followed by incubation with a primary antibody mouse anti Oct4 (#K1815, Santa Cruz, Santa Cruz, CA, USA 1:200) and mouse anti Tra-1-60 (#ab16288, Abcam, Cambridge, UK, 1:200), diluted in blocking solution and incubated in 4 °C overnight, washed and then incubated with a secondary antibody goat anti-mouse (#A21042 Alexa fluora 488, Thermo Scientific, Waltham, MA, USA, 1:500) for 1 h, and then counterstained with DAPI (D1306, Thermo Fisher Scientific, Waltham, MA, USA, 1:1000) for nucleus localization. Bright-field phase and fluorescence images of cells were captured using an Olympus IX51 inverted light microscope.

Quantification of immunohistochemistry results was performed using the open-source image analysis software Ilastik (version 1.4.1rc2) [21]. We employed the Pixel Classification tool to categorize the pixels into three distinct classes: background, unstained and stained cells. Post-training, the probability maps for each class across all images was extracted. If the total pixel values from the stained probability map was defined as S and from the unstained probability map as U, the staining ratio was calculated as S/(U + S). This measure is an estimate of the relative proportion of stained cells in each slide. Analyses were performed on data taken from three independent experiments. Error bars indicate s.e.m. Statistical significance was determined using Student’s *t* test, two sample unequal variance, one sided, *p < 0.05.

### Modeling the secondary protein structure

Theoretical structural models to predict APC secondary protein structure were created using the AlphaFold server, developed by DeepMind [22, 23].

### Statistical analysis

For all experiments, at least three independent experiments were carried out. p values were calculated using the Student’s *t* test, two sample unequal variance, one sided, and are represented as *p < 0.05 and **p < 0.01. All data are presented as the mean ± standard error.

## Results

**Fig. 1:**
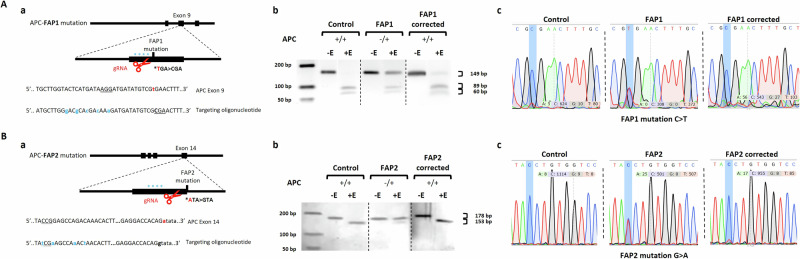
CRISPR gene editing correction of the APC germline mutation within FAP-hESC lines. **A** CRISPR gene editing correction of the APC germline mutation within FAP1-hESCs. **a** Strategy to correct the FAP1 mutation. Blue asterisks indicate silent mutations; FAP1 point mutation is marked with red. **b** Confirmation of APC mutation correction using RFLP. Corrected clones are expected to result in 89 bp and 60 bp for FAP1 and 153 bp for FAP2. Control (H9 line) and FAP(1 + 2) hESC lines served as positive and negative controls, respectively; cut with restriction enzyme: NurI (for FAP1) and BsaJI (for FAP2). **c** Sequencing results of the APC mutation region following CRISPR editing. The Hues13 hESC line served as a control for non-mutated APC; FAP1 for the heterozygous mutated APC and clone 37 for the corrected clone. **B** CRISPR gene editing correction of the APC germline mutation within FAP2-hESCs. **a** FAP2 mutation ata>gta is marked with red; silent mutations are marked in blue; protospacer adjacent motif (PAM) is underlined in purple. **b** Restriction fragment length polymorphism (RFLP) analysis of PCR products for APC mutation correction. Corrected clones are expected to result in 89 bp and 60 bp for FAP1 and 153 bp for FAP2. Control (H9 line) and FAP1 & FAP2 hESC lines served as controls. The restriction enzyme (E) NurI was used for FAP1, and BsaJI for FAP2. **c** Sequencing results of the APC mutation region following CRISPR editing. The H9 hESC line served as a control for non-mutated APC; FAP2 for the heterozygous mutated APC.

Previous results from our lab indicate differential competence to form organoids among the three FAP-hESC lines (FAP1, FAP2, and FAP3) harboring different germline mutations [17]. We have previously shown that FAP-hESCs carrying APC truncation mutations (FAP1 and FAP2) generated only a few cyst-like structures and cell aggregates of various shapes, while FAP3 generated complex and molecularly mature three-dimensional colonic structures. A correlation was found between the in vitro colon organoid maturation potential and the clinical severity of FAP in the carrier parent [17]. To prove that this organoid phenotype is specifically attributed to the single mutation in the APC gene, and not to any other genetic factor associated with the distinct genetic background of each FAP-hESC line, we established an APC mutation correction system based on CRISPR-Cas9. gRNAs were designed to target the mutation locus and a donor plasmid serves as a template for homolougs recombination for both FAP1 and FAP2. To avoid repeated targeting of the Cas9-gRNA, a silent-directed mutagenesis was designed at the PAM sequence of the donor-corrected plasmid (Fig. 1Aa and Fig. 1Ba). Following transfection of the CRISPR-Cas9 system into FAP1-hESCs, 63 clones were isolated, and 38 of them were validated by both RFLP and Sanger sequencing. In 10 clones the RFLP results hinted at a correction (Fig. 1Ab), of which five were confirmed by Sanger sequencing to align with the un-mutated sequence (Fig. 1Ac), resulting in 8% genome editing efficiency. The CRISPR/Cas9-based targeted genome editing of the APC germline mutation in FAP2 resulted in 64 clones of which 36 were screened by RFLP and only one demonstrated a complete correction by Sanger sequencing (Fig. Bb-c), resulting in 1.5% genome editing efficiency.

**Fig. 2:**
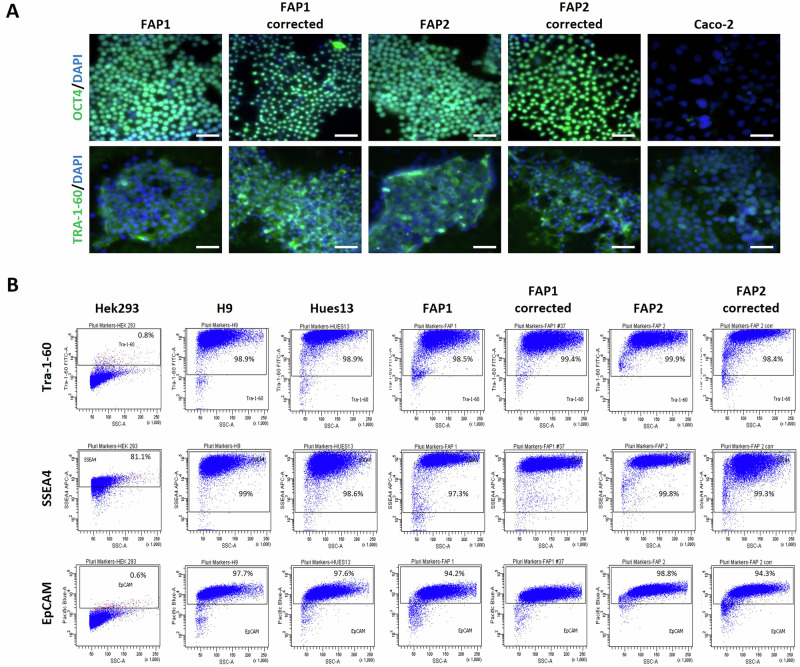
The CRISPR-based gene editing preserves the pluripotency of the corrected hESC lines. **A** Representative immunofluorescence images of the pluripotency markers OCT4 and TRA-1-60, in FAP1 & FAP2 corrected hESCs. **B** Flow cytometry analysis of the pluripotent stem cells markers, Tra-1-60, EpCAM, and SSEA4 in FAP1 & FAP2 corrected cells. HEK-293 cells served as a negative control and the WT-hESCs lines (H9 and Hues13) serve as a positive control. FAP1 & FAP2 are the heterozygotes APC mutated hESC lines and FAP1 corrected/FAP2 corrected are the CRISPER-edited corrected hESC lines.

To verify the preservation of pluripotency following CRISPR-mediated mutation correction, the corrected FAP1 and FAP2 clones were evaluated for the expression of pluripotent markers (SSEA4, TRA-1-60, EPCAM) by both immunofluorescence and FACS analysis (Fig. 2A, B). The results show that the cells maintained their stemness even after CRISPR-mediated mutation correction demonstrating >95% of pluripotent cells in both FAP1 & FAP2 corrected hESC lines.

**Fig. 3:**
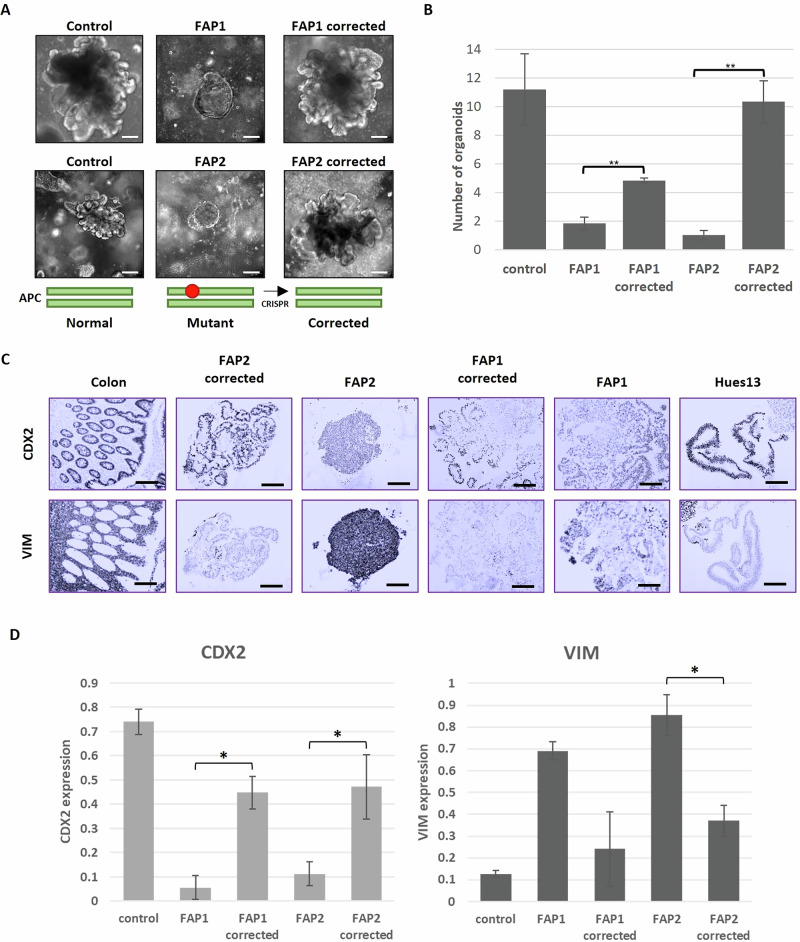
Correction of the APC germline mutation in FAP1-hESCs successfully rescue the colon organoid phenotype. **A** Representative images of day 45 colon organoids derived from hESCs. FAP1- and FAP2-hESC lines carry different germline APC mutations, and their corrected hESC lines are their isogenic control lines derived following CRISPR-mediated mutation correction. H9 served as a control hESC line with normal APC; Scale bar: 200 µm. **B** Quantification of Number of organoids per Matrigel droplet generated from each hESC line. Data are presented as Mean ± SEM from three independent experiments (2–3 Matrigel droplets per line). Student’s *t* test, two sample unequal variance, one sided, **p < 0.01. **C** Representative images of immunohistochemical analysis of colon epithelial markers CDX2 and the mesodermal marker vimentin (VIM) in day 45 organoids derived from FAP1- and FAP2-hESC and their isogenic controls. Normal colon tissue served as a positive control. Scale bars: 200 µM. **D** Quantification of CDX2 and VIM expression, by the Ilastik image ana lysis software. Analyses were performed on data taken from three independent experiments. Data are presented as Mean ± SEM. Student’s *t* test, two sample unequal variance, one sided, *p < 0.05.

In order to assess whether the APC mutation correction restores FAP1 and FAP2 ability to differentiate into mature colon organoids, the corrected FAP-hESC lines were then subjected to in vitro differentiation into colon organoids. Interestingly, the corrected clones developed into complex organoids resembling those derived from the control hESCs that are free of the APC mutation (H9 control hESC line; Fig. 3). In accordance, the number of differentiated complex organoids in the corrected cells was similar to that of the control (>5 differentiated organoids per Matrigel droplet), compared to only 1–2 that developed from the heterozygous APC/FAP1& FAP2 cells (Fig. 3B). Immunohistochemistry analysis shows high expression of the colon epithelial differentiation marker CDX2 in the corrected colon organoids, compared to their isogenic heterozygous mutant cells. In accordance, the corrected cells show reduced expression of the mesenchymal marker VIM compared to the mutant cells. These results demonstrate the colonic lineage characterization of cells within the organoids derived from the corrected cells, similar to the control (Fig. 3C, D). Collectively these results provide striking evidence that the heterozygote APC germline mutation is a primary driver for the in vitro simple organoids phenotype and the pre-tumorigenic phenotype of the cells.

### The effect of APC germline mutations on the predicted function of its encoded protein

**Fig. 4:**
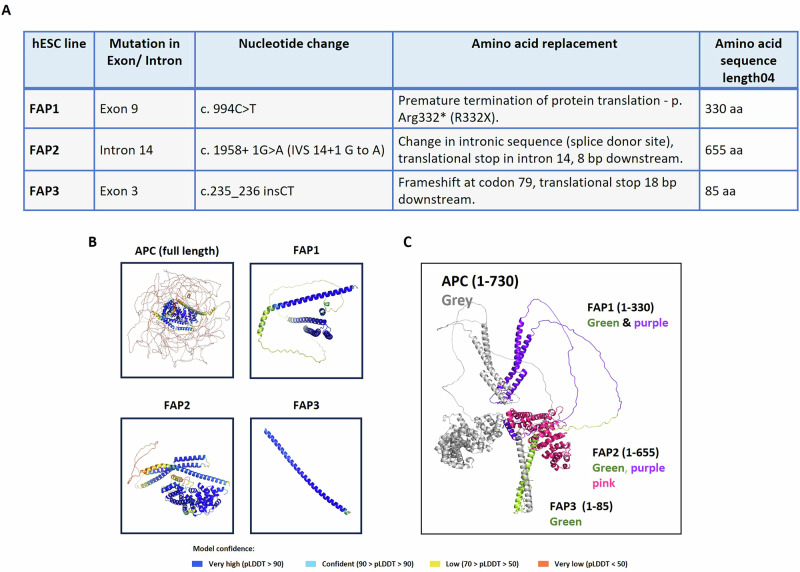
APC germline mutations and the secondary protein structure of the FAP-hESC lines. **A** Description of the type and location of APC germline mutations in each of the three FAP-hESC lines and their effect on the protein sequence. RYI – (R-Arginine, Y-Tyrosine, I-Isoleucine); LVISLE – (L-Leucine, V-Valine, I-Isoleucine, S-Serine, L-Leucine, E-Glutamine acid). **B** Model of the secondary protein structure of the full APC protein and the truncated forms of FAP1, FAP2 and FAP3. The AlphaFold2 model based on dLDDT of the APC full domain and the truncated forms of FAP1, FAP2 and FAP3. Model confidante is indicated in a color scale ranging from very low in orange to very high in blue. **C** The APC- FAP3, FAP1 and FAP2 model is showcased, delineating the various domains: APC is highlighted in gray, FAP1 in green and purple, FAP3 in green, and FAP2 in a combination of green, purple, and pink.

APC is considered as a scaffolding protein with several distinct domains that contribute to its various cellular interactions and functions. Our FAP-hESC lines (FAP1, FAP2, and FAP3) carry different heterozygous APC mutations (Fig. 4A) and exhibit different differentiation capacities in correlation with disease severity (Fig. 3 [17]). Variations in APC gene sequence, mutation type/location, and the resulting amino acid changes in FAP hESC lines likely contribute to the observed differences. In FAP1 the APC includes a stop codon in amino acid (aa) 332, resulting in a 330 aa protein. FAP2 harbors a mutation in intron 14, leading to a truncated protein of 655 aa. In FAP3, two nucleotides (CT) are inserted at position 235, leading to a frameshift mutation yielding a truncated 85 aa protein (Fig. 4A). In order to model the secondary protein structure of the full-length APC (FL-APC) protein and to provide insights into the spatial organization of the truncated proteins, the AlphaFold2 (AF) algorithm developed by DeepMind [22, 23] was utilized. FL-APC shows flexibility (low pLDDT scores) in intrinsically disordered regions, including the N-terminus (aa 1-131, relevant to FAP3) and aa 736-2483 (Fig. 4B). Conversely, the AF-generated models for FAP1, FAP2, and FAP3 exhibit predominantly high pLDDT scores, indicating a high level of confidence. Figure 4C depicts the full-length APC (gray) alongside structural models of truncated proteins from FAP lines: FAP3 (green), FAP1 (green and purple), and FAP2 (green, purple, and pink).

**Fig. 5:**
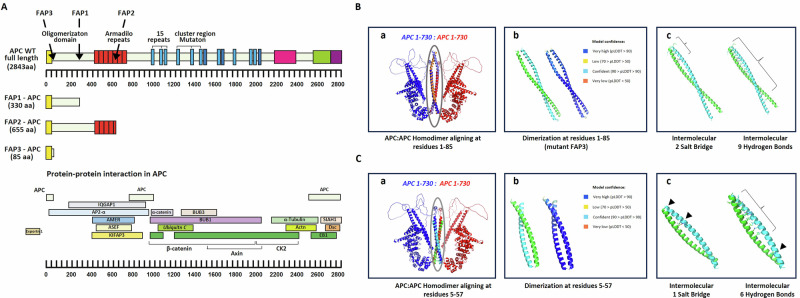
The three-dimensional structure of the APC homodimer. **A** Schematic primary structure of the resulting truncated APC forms characterizing FAP1, FAP2 and FAP3-hESC lines. The APC regions binding target proteins are indicated below. **B** The full APC-APC homodimer. **a** APC 1-730: APC 1-730 homodimer aligned on residues 1–85 where the mutation of FAP3 is located. **b** The two monomers are shown in green and light blue (left); The AlphaFold results show high model confident (right). **c** The homodimer structure is held is shape by either intermolecular salt bridge (left; Gln12 & Lys17; or Glu19 & Arg 24), and/or intermolecular hydrogen bonds (right; Ala4-Tyr6-Leu9; Leu10-Gln12; Val13-Leu16; Lys17-Asn20; Leu23-Arg24; Leu27-Asn30; His33-Leu34; Leu37-Leu48; Gln65-Leu72). **C**, **a** APC 1-730: APC 1-730 homodimer aligned on residues 5-57 where the mutation of FAP3 is located. **b** The two monomers are shown in green and light blue (left); The AlphaFold results show high model confident (right). **c** The homodimer structure is held is shaped by either intermolecular salt bridge (left; Glu20 & Arg26) and/or intermolecular contacts involving the indicated residues (right; Leu9-Leu10-Gln12; Val13-Leu16; Glu19-Asn20; Leu23-Arg24; Leu27-Asn30; His33-Leu34-Leu48).

Protein dimerization is crucial for various cellular processes. Proteins can form homodimers or heterodimers and disruptions in these interactions can lead to various diseases. We investigated both homodimerization of native APC and the heterodimers formed with truncated proteins resulting from the three FAP mutations. Figure 5A depicts the schematic primary structure of the APC protein, highlighting its key domains: (1) The heptad repeats (yellow) located in the N-terminus oligomerization domain. Retention of amino acids 5–57 is essential for APC oligomerization [7, 24]; (2) The Armadillo region (red) consists of seven repeats, highly conserved and shows a high degree of homology to a similar area in β-catenin.; (3) The three 15–20 amino acid signature repeats (blue) binds β-catenin; (4) The Multiple cluster region (MCR) is where most of the second mutations occur. The truncated FAP1, FAP2 and FAP3 proteins retain only parts of the N-terminal domain. The AF algorithm predicts high-confidence homodimerization for full-length APC through 1-730aa. FAP3’s N-terminus seems critical, potentially involving residues 1-85 or even just 5-57 (Fig. 5B, C) The highly confident FAP3 dimerization at residues 1-85 generates numerous intermolecular connections of Salt Bridges (SB) in the N-terminal and hydrogen bridges scattered across the dimerization (Fig. 5B). Indeed our further analysis demonstrates dimerization with very high confidence at this region through intermolecular salt bridge and hydrogen bonds (Fig. 5C).

**Fig. 6:**
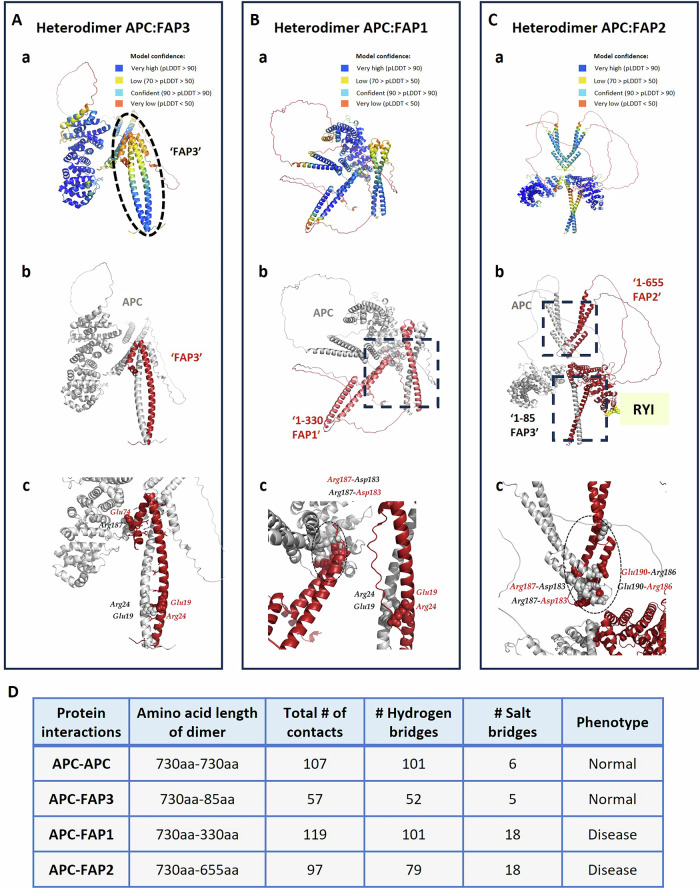
The APC heterodimers. **A** The APC:FAP3 heterodimer. **a** The dotted circle marks the ‘FAP3’ domain. The AlphaFold2 results show the confidence of APC: FAP3 heterodimer, at least in most parts of the protein. **b**, **c** The two monomers are shown in red (FAP3) and gray (APC). Effect of the mutation on dimer formation of APC: FAP3, based on chemical bonds at the truncated protein. Salt bridges between APC and FAP3, the interacting residues are indicated: Arg24-Glu19; Glu19-Arg24; Arg187-Glu74. **B** The APC:FAP1 heterodimer. **a** The AlphaFold2 results show high confidence in the model. **b** The two monomers are shown: in pink (1-330 FAP1 truncated protein) and gray (full APC). **c** The depicted images represent magnified views of the enclosed regions of interaction. Salt bridges between FAP1- APC are shown in balls: Arg24-Glu19; Glu19-Arg24; Arg187-Asp183; Asp183-Arg187. **C** The APC:FAP2 heterodimer. **a** The AlphaFold2 shows the confidence of the model. **b** The two monomers are shown: in red (FAP2) and gray (APC). The three additional residues RYI (R-Arginine, Y-Tyrosine, I-IsoLeucine) characterizing the truncated FAP2 protein are shown in yellow. The residues involved with heterodimer formations are from FAP3 and FAP1 but not beyond residue 330. **c** The depicted images represent magnified views of the enclosed regions of interaction and the salt bridge formation between FAP2: APC is illustrated, with the interacting residues indicated. **D** Effect of salt bridges on the heterodimer structure—a table summarizing protein length, number of contacts within the dimer and the type & number of contacts within the APC homodimer/heterodimers.

To predict full-length APC heterodimerization with truncated FAP proteins, we generated structural models (APC-FAP1/2/3) using AlphaFold models and find possible chemical bonds that may play a role in stabilizing the heterodimer (Fig. 6). Notably, the first five suggesting structural models of AF for each of these three heterodimers exhibited distinct orientations (data not shown). APC-FAP3 heterodimer shows high confidence in FAP3’s N-terminus, decreasing gradually towards the end of the dimerization interface (Fig. 6Aa). APC-FAP3 heterodimer exhibits 57 contacts, including 52 hydrogen bonds along the α-helix and 5 salt bonds, notably Glu74-Arg187 (Fig. 6A, D). The model for APC: FAP1 predominantly displays high pLDDT scores, indicative of a considerable level of confidence (Fig. 6B). FAP1 mutation creates a 119-contact heterodimer with full APC. Beyond shared salt bridges and hydrogen bonds in the area of FAP3, APC-FAP1 predicts an additional salt and hydrogen bond such as Arg187-Asp183 salt bond. FAP2 (1-655) creates a 97-contact heterodimer with full APC, and its mutation that leads to additional residues (RYI), do not affect heterodimerization (Fig. 6C). Structural analysis reveals FAP1/2 form heterodimers with FL-APC via significantly more salt bridges (18/18 respectively) compared to APC homodimers [6] and FAP3 [5]; (Fig. 6D; Supplementary Table 1). These findings suggest that the APC mutations in FAP1 and FAP2 likely underlie their inability to form complex colon organoids, unlike control and FAP3 lines.

We then examined the C-termini of each truncated protein of FAP1, FAP2, and FAP3 to identify potential charges that may impact protein interactions with APC or other proteins. FAP1 has a stop codon in amino acid 332, but no potential charges or any other interaction were detected (Supplementary Fig. 1A). In accordance, The additional amino acids in FAP3 do not affect its structure or seem to produce interactions with other proteins (supplementary Fig. 1C). However, In the fragmented FAP2 protein, in addition to the salt bridge situated at the truncated terminus, supplementary salt bridges are identified along the protein (supplementary Fig. 1B). Interestingly, salt bridges are also formed in the sequence included in FAP1, apparently as a result of the addition of 3 residues and the size of the protein (655 aa compared to 330 aa of FAP1), which causes a different spatial organization of FAP2.

**Fig. 7:**
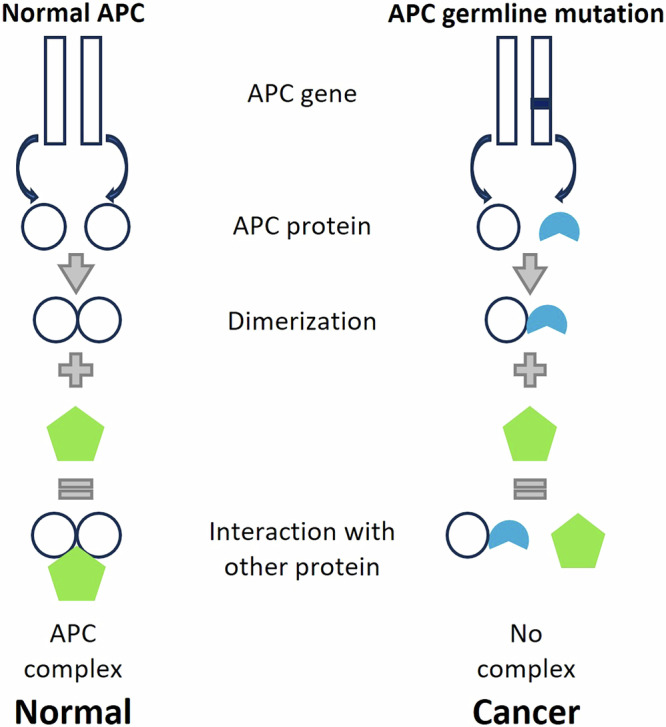
Dominant-negative effect of truncated APC through heterodimerization. **Left:** Full-length APC protein (white) forms homodimers and binds to the β-catenin destruction complex (green), leading to normal colon phenotype. **Right:** Truncated APC protein (blue) binds to a full-length APC (white) forming a heterodimer. However, this binding disrupts the interaction with the β-catenin destruction complex, leading to abnormal function and ultimately tumor formation.

Our findings propose a model linking specific heterozygous APC mutations to colon organoid dysfunction in vitro and tumorigenesis in patients (Fig. 7). In individuals with wild-type APC genes, the two functional proteins undergo homodimerization and interact with the β-catenin destruction complex to enable its proper function, a critical step in tumor suppression. Conversely, germline mutations in the APC gene, as observed in FAP1 and FAP2, lead to the translation of a truncated protein from the mutated allele, which forms a heterodimer with the second full-length APC allele. Increased salt bridges in FAP1/2 heterodimers disrupt APC function compared to normal homodimers, likely due to a dominant-negative effect of the truncated protein. This disrupts β-catenin destruction complex activity, promoting tumor formation.

## Discussion

FAP is a hereditary condition with a high risk for CRC. Our unique collection of FAP-hESC lines with distinct heterozygous APC mutations offer a unique human in vitro model for studying early FAP progression and severity. In this study, we successfully generated isogenic control lines utilizing CRISPR technology for our two FAP hESC lines (FAP1, FAP2), which exhibit a severe disease phenotype. Strikingly, these isogenic control lines demonstrate the remarkable ability to form complex and well- developed colon organoids expressing intestinal markers like those observed in normal expressing APC organoids and in contrast to the inability of the parental heterozygous FAP1 and FAP2 lines to generate colon organoids. These findings provide further evidence of the early molecular events associated with germline APC mutations in colon cancer, offering additional insights into disease progression.

As APC regulates cell growth and division, a single mutated copy disrupts its function, leading to increased β-catenin levels and nuclear accumulation, ultimately activating the oncogenic Wnt signaling pathway and promoting chromosomal instability [25, 26]. A single germline mutation in the APC gene, although insufficient for complete tumorigenesis, predisposes individuals to colon cancer by initiating early events associated with uncontrolled cellular proliferation. A subsequent mutation in the remaining wild-type allele (second hit) is necessary for full malignant transformation [27, 28].

Like FAP, many other diseases start with a single germline mutation affecting genes involved in cell growth regulation. For instance, Hereditary Breast and Ovarian Cancer (HBOC) is associated with mutations in BRCA1/2 [29, 30], Von Hippel-Lindau (VHL) syndrome is associated with mutations in the VHL gene [31, 32], and Hereditary Retinoblastoma is associated with mutations in the RB1 gene [33, 34]. However, the precise mechanisms by which germline mutations initiate the early stages of tumorigenesis remain unclear, even in these diseases.

Previous studies have demonstrated a genotype-phenotype correlation in FAP, where the location of APC germline mutations may influence disease severity and the age at which CRC develops [29, 35–37]. The first documented link is between truncating mutations within codons 1250 and 1464 in the APC gene and a severe FAP disease characterized by an exceptionally high number of polyps, termed Profuse FAP [35, 38]. Mutations near the 5’ end of APC (upstream to codon 157) or downstream codon 1595, including the alternatively spliced region of exon 9, are correlated with a milder form of FAP termed Attenuated FAP (AFAP), characterized by less than 100 colorectal adenomas [39–42]. FAP patients with mutations in other APC gene regions tend to exhibit an intermediate phenotype, characterized by polyp numbers between the hundreds and thousands [29, 43].

This study reveals insights into the spatial organization of truncated APC proteins arising from three disease-associated FAP germline mutations, and how these alterations impact the structure of the resulting APC heterodimers. Protein dimerization plays crucial roles in regulating various physiological processes in the body by forming homo-, hetero-, or oligomerization that regulate cellular processes. Any deregulation of these processes may result in a disease state. In this study, we employed the AlphaFold2 algorithm to predict the secondary structures of the full-length APC protein, its truncated variants, and their respective heterodimers. Our analysis of novel salt bridges formed within the heterodimers and the potential impact of altered charge distribution on protein interactions suggests that truncated APC proteins exert a dominant-negative effect, disrupting normal APC function by impairing its interaction with the β-catenin destruction complex, ultimately leading to tumor formation.

Predicting the structure of large proteins like APC poses a challenge due to their size and complexity. Current efforts have focused on applying AlphaFold models to predict the structure of the N-terminal region of the human APC protein [44, 45]. Though AlphaFill modeled the human APC protein’s N-terminus, intrinsically disordered regions remain a challenge due to low confidence in their structure and high dynamics, suggesting flexible binding sites for diverse partners [46]. Unraveling the 3D structure of oncogenic proteins like APC is crucial. This knowledge unlocks their functions, pinpoints key regions for interaction, and reveals how they change shape in response to signals. It also paves the way for designing drugs that specifically target these proteins. These inhibitors can disrupt aberrant signaling pathways in neoplastic cells by specifically binding to functional domains or key regions within the protein.

APC acts as a multi-domain scaffold protein with distinct binding sites for various targets. Key domains interact with β-catenin and Axin, regulating β-catenin degradation [10, 47–49]. Additionally, APC interacts with proteins like Asef, IQGAP1, and KIFAP3, influencing cell migration, adhesion, and transport [10, 50–53].

The APC protein functions as a dimer, facilitated by specific structural domains critical for its role in the β-catenin destruction complex. Its N-terminal region contains heptad repeats, forming coiled-coil structures that promote homodimerization between APC molecules [7, 24, 54]. Furthermore, APC has multiple domains that facilitate protein–protein interactions including the armadillo repeat region, the 15 and 20 amino acid repeats (β-catenin binding), SAMP repeats (Axin binding), a basic domain (microtubule binding), an end binding protein (EB1) interaction domain, and a PDZ binding motif at the carboxy terminus. [52]. In Wnt signaling, APC dimers act as scaffolds for assembling the destruction complex, which includes Axin, GSK-3, and CK1. This complex promotes the phosphorylation and subsequent degradation of β-catenin, thereby regulating Wnt signaling and preventing uncontrolled cell proliferation [55–57]. Truncated APC proteins containing heptad and Armadillo repeats can still interact with wild-type APC, to form different types of heterodimers potentially disrupting its normal function and contributing to tumorigenesis [58, 59]. Our results on predicting the secondary structures of FAP1,2,3 truncated variants, which harbor dimer formation repeats, indicate that FAP1 and 2 bind the FL-APC and interrupt APC’s scaffold function, preventing its interactions with target proteins relevant for its proper function.

Traditionally, complete loss-of-function (LOF) mutations in tumor suppressor genes are expected to elicit a more severe phenotypic effect than truncating mutations that retain some residual protein function [60]. While FAP1 & 2 cells with truncated APC display severe organoid phenotypes as expected, FAP3 cells with an even shorter protein surprisingly formed normal colon organoids. Our findings thus support the proposed dominant-negative effect exerted by truncated APC proteins in FAP1 and FAP2 lines, which retain at least the first 171 amino acids of the protein [3, 61, 62]. In contrast, FAP3, while retaining the oligomerization domain, likely lacks a dominant-negative effect due to its significantly smaller size (85 amino acids). Our protein structure and interaction analyses suggest a dominant-negative mechanism for specific APC variants: the increased size of FAP1 & FAP2 likely grants them greater flexibility than the FL-APC and the short FAP3 proteins, potentially allowing them to access internal binding sites within heterodimers that are inaccessible in homodimers. These stronger intermolecular interactions (salt bridges and hydrogen bonds) with FL-APC create highly stable heterodimers, disrupting normal APC function by blocking protein interactions and assembly in the β-catenin destruction complex.

Our findings demonstrate a clear link between the type of APC germline mutation and suggest the mechanism by which the specific truncated proteins mediate colon organoid formation capacity, further emphasizing the genotype-phenotype correlation in FAP. Elucidating these relationships holds promise for deciphering disease progression and paving the way for personalised therapeutic approaches tailored to individual patient mutations.

## Supplementary information

Supplementary Table 1

Supl. Fig. 1

Supl. Fig. 1

## Data Availability

All data generated or analyzed during this study are included in this published article and its supplementary information files.
