# Supplementary figures and images for: Predicting colorectal cancer risk in FAP patients using patient-specific organoids
Source: Cancer Gene Ther. 2025 Jul 22;32(9):997–1007. doi: 10.1038/s41417-025-00923-7 (PMC12396958; doi:10.1038/s41417-025-00923-7)

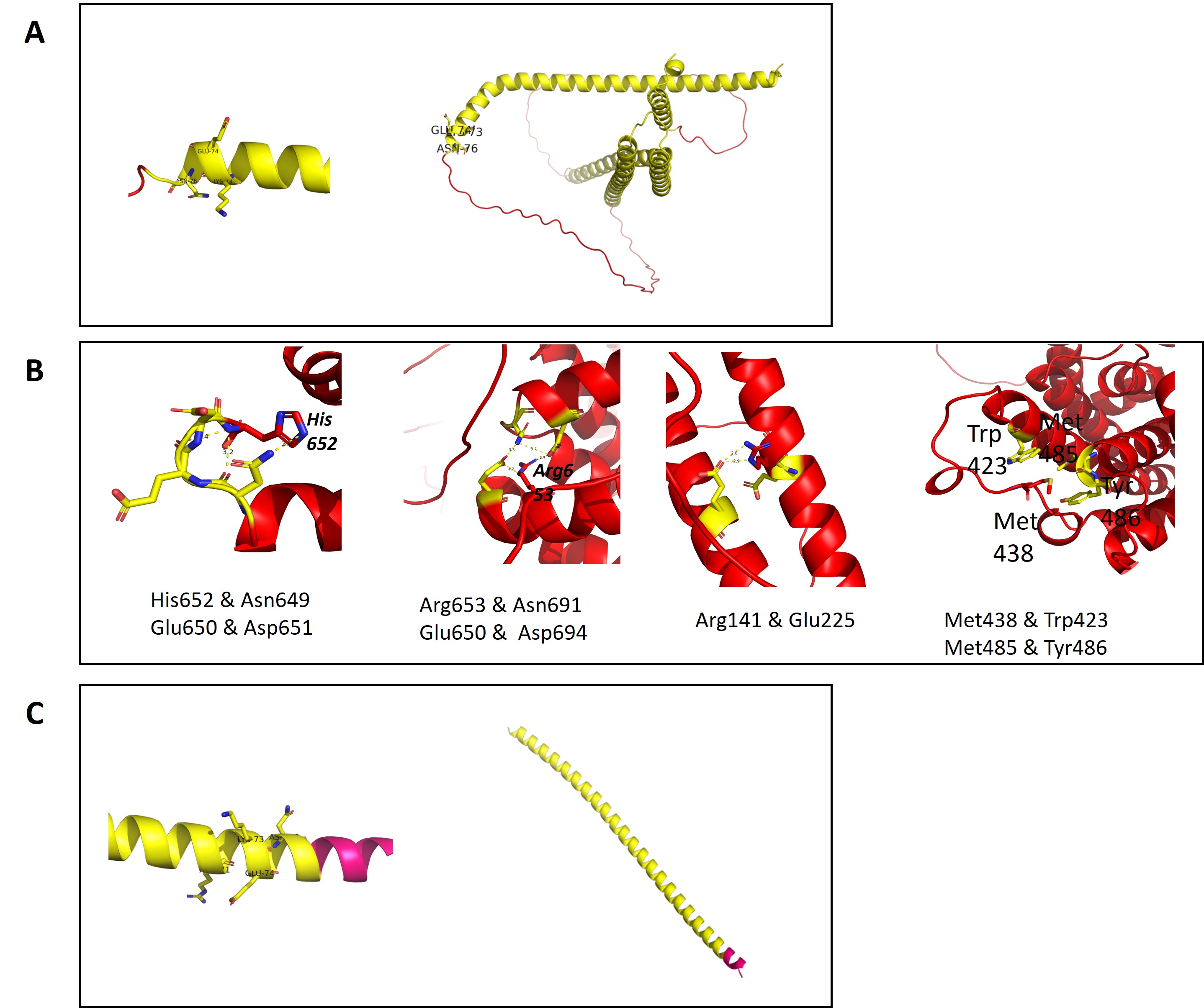

Supplement: Supplementary file 2 — Supl. Fig. 1 [file 41417_2025_923_MOESM2_ESM.jpg]
