# Supplementary material for: Predicting colorectal cancer risk in FAP patients using patient-specific organoids
Source: Cancer Gene Ther. 2025 Jul 22;32(9):997–1007. doi: 10.1038/s41417-025-00923-7 (PMC12396958; doi:10.1038/s41417-025-00923-7)
Supplement: Supplementary file 3 — Supl. Fig. 1 [file 41417_2025_923_MOESM3_ESM.docx]

**Supl. Fig. 1**: **Effect of the mutation on dimer formation (based on chemical bonds at the truncated protein)**

**(A)** FAP1 - The resulting residues do not interact with APC. **(B)** FAP2 - Intramolecular salt bridge interaction between the indicated residues is shown. **(C)** FAP3 - This change in protein sequence does not predict any structural difference.
